# Health-Related Behaviors and Effectiveness of Trivalent Inactivated versus Live Attenuated Influenza Vaccine in Preventing Influenza-Like Illness among Young Adults

**DOI:** 10.1371/journal.pone.0102154

**Published:** 2014-07-11

**Authors:** Tabitha Woolpert, Christopher J. Phillips, Carter Sevick, Nancy F. Crum-Cianflone, Patrick J. Blair, Dennis Faix

**Affiliations:** 1 Operational Infectious Diseases, Naval Health Research Center, San Diego, California, United States of America; 2 Deployment Health Research, Naval Health Research Center, San Diego, California, United States of America; The University of Adelaide, Australia

## Abstract

**Background:**

Vaccination is the preferred preventive strategy against influenza. Though health behaviors are known to affect immunity and vaccine delivery modes utilize different immune processes, data regarding the preferred influenza vaccine type among adults endorsing specific health-related behaviors (alcohol use, tobacco use, and exercise level) are limited.

**Methods:**

The relative effectiveness of two currently available influenza vaccines were compared for prevention of influenza-like illness during 2 well-matched influenza seasons (2006/2007, 2008/2009) among US military personnel aged 18–49 years. Relative vaccine effectiveness was compared between those self-reporting and not reporting recent smoking history and potential alcohol problem, and by exercise level using Cox proportional hazard modeling adjusted for sociodemographic and military factors, geographic area, and other health behaviors.

**Results:**

28,929 vaccination events and 3936 influenza-like illness events over both influenza seasons were studied. Of subjects, 27.5% were smokers, 7.7% had a potential alcohol-related problem, 10.5% reported minimal exercise, and 4.4% reported high exercise levels. Overall, the risk of influenza-like illness did not significantly differ between live attenuated and trivalent inactivated influenza vaccine recipients (hazard ratio, 0.98; 95% confidence interval, 0.90–1.06). In the final adjusted model, the relative effectiveness of the 2 vaccine types did not differ by smoking status (*p* = 0.10), alcohol status (*p* = 0.21), or activity level (*p* = 0.11).

**Conclusions:**

Live attenuated and trivalent inactivated influenza vaccines were similarly effective in preventing influenza-like illness among young adults and did not differ by health-related behavior status. Influenza vaccine efforts should continue to focus simply on delivering vaccine.

## Introduction

Certain risky health behaviors, such as tobacco use and significant alcohol consumption, are known to affect disease risk by impairing the immune response. Studies have documented impaired mucosal immunity among smokers compared with nonsmokers [1] and negative cellular and innate immune system effects among excessive alcohol consumers [2]–[10], providing a basis for a differential vaccine effectiveness of the available influenza vaccines that use different delivery methods. Since smoking and alcohol use are associated with a higher risk of respiratory infections including influenza, defining optimal vaccine strategies among these groups is important for both patient care and public health strategies [11]–[13].

Vaccination against influenza virus has been a common practice since the vaccine's development in 1945 [14], and is the single most effective prevention strategy [15]–[17]. Since it is expected that the majority of individuals worldwide will remain susceptible to one or more circulating influenza strains at any given time, continued understanding of optimal vaccine practices is essential. Two of the influenza vaccine types currently licensed in the United States include the trivalent inactivated influenza vaccine (TIV), an older formulation, and live, attenuated influenza vaccine (LAIV), licensed since 2003. TIV is prepared by killing influenza viruses and isolating subvirion or purified surface antigens to create “split” or subunit vaccines [16], while LAIV comprises attenuated live viruses. Vaccine antigens contained in LAIV and TIV are presented to an individual's immune system through markedly different routes: TIV is injected, causing rapid distribution into the bloodstream and induction of a humoral response, while LAIV virions are delivered to the nasopharyngeal epithelial cells where they appear to induce both humoral (mucosal IgA antibody) and cell-mediated immune responses [18].

Because various immune mechanisms are involved to differing degrees between the two vaccine methods, the immune response induced by each could differ among individuals with altered immunity. In addition to smoking and alcohol use, intense exercise may alter immunity through various mechanisms including transient reduced salivary IgA levels [19]–[24], providing a basis for a potential differential vaccine response to LAIV versus TIV. Likewise, though its immune effects are not well established, a sedentary lifestyle may result in adverse health consequences, while exercise has been shown to boost immunity [25]–[27]. Hence, differential vaccine responses by exercise level are plausible.

Given the high prevalence of adverse health-related behaviors in the general population [28], a statistical difference in vaccine effectiveness between LAIV and TIV in these risk groups would likely correspond to a significant influenza disease burden that could be eliminated with tailored vaccine protocols. The risk of influenza-like illness (ILI) by vaccine type received among smokers, those with a potential alcohol problem, and those reporting varying levels of exercise was evaluated in this study.

## Methods

### Study Population and Data Sources

A retrospective cohort study was conducted during 2012–2013 among US military members who completed a health survey for a large prospective Department of Defense study (Millennium Cohort Study) [29] within 2 years of influenza vaccination. Subjects were active-duty members stationed in the United States at the beginning of the influenza season who received influenza vaccination during the 2006/2007 and/or 2008/2009 seasons. These seasons were chosen because vaccine strains were well matched to circulating influenza strains, according to surveillance data published by the Centers for Disease Control and Prevention (CDC) [30]–[32]. Although 66% of influenza A (H1N1) viruses were found to be similar to the vaccine strain, the 2007/2008 influenza season was excluded as 77% of influenza A (H3N2) and 98% of B viruses were poorly matched [33]. All participants were aged 18–49 years at the time of vaccination since LAIV use is limited to adults ≤49 years of age. Exclusion criteria included a diagnosis of asthma, chronic bronchitis, emphysema, history of shortness of breath, diabetes mellitus, HIV infection, or pregnancy, since LAIV use may have been contraindicated and these conditions may result in suboptimal vaccine responses. Data on preexisting medical diagnoses were obtained from the Millennium Cohort survey and medical records. Additionally, pharmacy records (Pharmacy Data Transaction Service) were used to exclude subjects receiving medications for diabetes mellitus or HIV. Non–active duty military personnel, personnel deployed at the time of study, and members stationed on ships or overseas were excluded because these groups may be exposed to influenza strains not covered in the vaccine and outcomes of interest may not be captured in existing databases. Additionally, military recruits were excluded given their unique risks for respiratory illnesses [34]–[37].

The type and date of influenza vaccinations were obtained from military records maintained by the Defense Manpower Data Center (DMDC). Annual influenza vaccination is mandatory for active-duty members, though the type of vaccine (TIV or LAIV) is often based on available supply. The study was approved by the Institutional Review Board at the Naval Health Research Center (protocol NHRC.2010.0027), in accordance with 32 CFR §219. l 16(d) and classified as minimal risk. The IRB waived the requirement to obtain informed consent because it is impractical with numerous record holders that are geographically widely-dispersed, and the decision to waive informed consent will not adversely affect the rights and welfare of the record holders of this study population.

### Study Outcome

The outcome of interest was ILI, chosen because laboratory testing for influenza virus is not commonly employed and because ILI has both medical and occupational significance. The diagnosis of ILI was determined by review of *International Classification of Diseases, 9th Revision* (ICD-9) codes in the Military Health System Data Repository, which contains all outpatient and inpatient encounters from military treatment facilities and care provided by TRICARE network providers. An ILI case was defined by the presence of specific ICD-9 codes (Table 1) shown in previous studies to be related to culture-confirmed influenza in military service members [38], [39]. Independent of the presence of these ICD-9 codes, anti-influenza medication dispensing (i.e., oseltamivir, zanamivir, amantadine, or rimantadine) documented in pharmacy records was also considered to represent a case.

**Table 1:** ICD-9 Codes Used To Identify Influenza-like Illness.

ICD-9 Code	Description
079.99	Viral infection, NOS
382.9	Otitis media, NOS
460.0	Nasopharyngitis, acute
461.9	Acute sinusitis, unspecified
465.8	Upper respiratory infection, other multiple sites
465.9	Upper respiratory infection, acute NOS
466.0	Bronchitis and bronchiolitis, acute
486.0	Pneumonia, organism unspecified
487.0	Influenza with pneumonia
487.1	Influenza with other respiratory manifestations
487.8	Influenza with other manifestations
490.0	Bronchitis, not specified as acute or chronic
780.6	Fever
786.2	Cough

Outcomes were captured beginning September 1 of each season, and only the first ILI event per subject in each season was considered in the analyses. Subjects with an event during the current influenza season occurring prior to 14 days post vaccination were excluded from analyses to ensure that only vaccinated individuals with adequate time for development of an immune response and no recent influenza infection were studied. Subjects were followed from the time of influenza vaccination (administered September 1–November 30 of each season) until the end of the influenza season (May 19, 2007, and March 28, 2009; the latter season truncated given the emergence of the pandemic H1N1 strain) for occurrence of an ILI outcome, or censoring event [32], [40]. Individuals were right-censored if they deployed by land or aboard ship or transferred outside the continental United States. Subjects could contribute to both influenza seasons if they received vaccine and met the inclusion/exclusion criteria during each season.

### Exposures of Interest

Three specific exposures of interest were evaluated: recent smoking history, potential alcohol problem, and exercise level. Exposure data were obtained from self-reported survey information from the Millennium Cohort Study. Recent smoking history was defined as reporting both ≥100 cigarettes in a lifetime and cigarette use within the last year. Potential alcohol problems were defined as an affirmative answer to 1 or more of the 5 alcohol questions on the Patient Health Questionnaire [41], which represents a positive screen for alcohol abuse [42]. Exercise was categorized based on the current CDC physical activity recommendations [43]: the number of days exercised per week at a moderate level multiplied by time (in minutes) plus twice the number of days per week of vigorous exercise multiplied by time. If the result was <150 minutes then the exercise level was minimal, and if 6–7 days per week of vigorous activity was reported then the level was high; otherwise, the classification was moderate. Those with missing exercise activity data were denoted as unknown.

### Covariates

Information on sociodemographic and military-related characteristics were obtained from DMDC and included age, sex, self-reported race/ethnicity, marital status, service branch, military pay grade (enlisted, officer), duty station (coded into four geographic areas), occupation, and deployment history.

### Statistical Analyses

Descriptive statistics are presented as numbers (percentages) for categorical variables and medians (interquartile range, IQR) for continuous variables. The univariable associations between the covariates and vaccine type were assessed using logistic regression (for categorical variables) and the Wilcoxon rank-sum test for age (continuous). Crude incidence rates of ILI per 1000 persons per season were compared between the LAIV- and TIV-immunized groups. Cox proportional hazard modeling was used to evaluate the unadjusted and adjusted risk (time to event) for ILI by vaccine type (LAIV vs TIV) as well as other covariates of interest (sociodemographic, military, geographic, and behavioral factors). To test the hypothesis that relative vaccine effectiveness is differential by specific health behavior (smoking, alcohol, and exercise level), an interaction term with vaccine type was added to the model for each behavior. In addition, interactions were evaluated between vaccine type and 3 covariates: sex, service branch, and influenza season. In order to adjust for the potential effects of individuals contributing to multiple seasons, a robust variance estimator was used. To account for individuals receiving the vaccination at different times in relation to the influenza season, a left-truncated model was developed starting survival time at September 1 of the influenza season and truncating on date of vaccination plus 14 days. Follow-up time was measured from September 1 of each influenza season until an outcome was observed or the censor date. A final multivariable model was derived using a backward, stepwise approach and was adjusted for covariates significantly associated with the outcome (*p*<0.05) or that confounded the relationship between vaccine type and ILI by ≥10%. Results of the Cox models are presented as hazard ratios (HRs) and 95% confidence intervals (CIs). All statistical analyses were performed using SAS software, version 9.3 (SAS Institute, Inc., Cary, NC).

## Results

Overall, 28,929 vaccination events were studied during the 2 influenza seasons (2006/2007 and 2008/2009), including 22,734 (79%) LAIV and 6195 (21%) TIV administrations. The median age of the cohort was 27 years (IQR, 23–34), 73% were white, and 73% male. Regarding health-related behaviors, 27.5% subjects reported a recent smoking history and 7.7% a potential alcohol problem. Exercise levels were distributed as follows: 10.5% minimal, 70.3% moderate, 4.4% high, and 14.7% unknown (Table 2).

**Table 2:** Population Characteristics^a^ Among US Service Members, 18–49 Years of Age, During Two Influenza Seasons (2006/2007, 2008/2009).

	Total Study	2006/2007		2008/2009		Combined Seasons	
Characteristic	Population	TIV	LAIV	TIV	LAIV	TIV	LAIV
Number	28,929	2905	10,316	3290	12,418	6195	22,734
ILI event	3936 (13.6)	383 (13.2)	1477 (14.3)	404 (12.3)	1672 (13.5)	787 (12.7)	3149 (13.9)
Recent smoking history	7955 (27.5)	776 (26.7)	2814 (27.3)	941 (28.6)	3424 (27.6)	1717 (27.7)	6238 (27.4)
Potential alcohol problem	2221 (7.7)	292 (10.1)	760 (7.4)	274 (8.3)	895 (7.2)	566 (9.1)	1655 (7.3)
Exercise level							
Minimal	3039 (10.5)	345 (11.9)	1062 (10.3)	360 (10.9)	1272 (10.2)	705 (11.4)	2334 (10.3)
Moderate	20,345 (70.3)	2021 (69.6)	7243 (70.2)	2292 (69.7)	8789 (70.8)	4313 (69.6)	16,032 (70.5)
High	1283 (4.4)	139 (4.8)	461 (4.5)	137 (4.2)	546 (4.4)	276 (4.5)	1007 (4.4)
Unknown	4262 (14.7)	400 (13.8)	1550 (15.0)	501 (15.2)	1811 (14.6)	901 (14.5)	3361 (14.8)
Age, years	27 (23–34)	25 (22–32)	25 (22–33)	28 (24–35)	27 (24–35)	27 (23–34)	27 (23–34)
Sex							
Male	21,003 (72.6)	2046 (70.4)	7537 (73.1)	2241 (68.1)	9179 (73.9)	4287 (69.2)	16,716 (73.5)
Female	7926 (27.4)	859 (29.6)	2779 (26.9)	1049 (31.9)	3239 (26.1)	1908 (30.8)	6018 (26.5)
Race/ethnicity							
White/non-Hispanic	21,090 (72.9)	2195 (75.6)	7520 (72.9)	2345 (71.3)	9030 (72.7)	4540 (73.3)	16,550 (72.8)
Black/non-Hispanic	3620 (12.5)	286 (9.8)	1305 (12.7)	446 (13.6)	1583 (12.7)	732 (11.8)	2888 (12.7)
Hispanic	2077 (7.2)	206 (7.1)	714 (6.9)	261 (7.9)	896 (7.2)	467 (7.5)	1610 (7.1)
Other/unknown	2142 (7.4)	218 (7.5)	777 (7.5)	238 (7.2)	909 (7.3)	456 (7.4)	1686 (7.4)
Marital status							
Married	17,857 (61.7)	1605 (55.2)	5847 (56.7)	2233 (67.9)	8172 (65.8)	3838 (62.0)	14,019 (61.7)
Other	11,072 (38.3	1300 (44.8)	4469 (43.3)	1057 (32.1)	4246 (34.2)	2357 (38.1)	8715 (38.3)
Service branch							
Air Force	14,291 (49.4)	1168 (40.2)	5469 (53.0)	1215 (36.9)	6439 (51.9)	2383 (38.5)	11,908 (52.4)
Army	9405 (32.5)	651 (22.4)	3639 (35.3)	923 (28.1)	4192 (33.8)	1574 (25.4)	7831 (34.5)
Marine Corps	2974 (10.3)	774 (26.6)	679 (6.6)	651 (19.8)	870 (7.0)	1425 (23.0)	1549 (6.8)
Navy	2259 (7.8)	312 (10.7)	529 (5.1)	501 (15.2)	917 (7.4)	813 (13.1)	1446 (6.4)
Rank							
Enlisted	21,931 (75.8)	2117 (72.9)	7914 (76.7)	2421 (73.6)	9479 (76.3)	4538 (73.3)	17,393 (76.5)
Officer	6998 (24.2)	788 (27.1)	2402 (23.3)	869 (26.4)	2939 (23.7)	1657 (26.8)	5341 (23.5)
Occupation							
Combat	5231 (18.1)	478 (16.5)	1821 (17.7)	542 (16.5)	2390 (19.2)	1020 (16.5)	4211 (18.5)
Health care	4280 (14.8)	592 (20.4)	1449 (14.0)	724 (22.0)	1515 (12.2)	1316 (21.2)	2964 (13.0)
Other	19,418 (67.1)	1835 (63.2)	7046 (68.3)	2024 (61.5)	8513 (68.6)	3859 (62.3)	15,559 (68.4)
US geographic area							
Southwest	8746 (30.2)	1101 (37.9)	2997 (29.1)	1348 (41.0)	3300 (26.6)	2449 (39.5)	6297 (27.7)
Southeast	8992 (31.1)	966 (33.3)	3322 (32.2)	954 (29.0)	3750 (30.2)	1920 (31.0)	7072 (31.1)
Northeast	6367 (22.0)	537 (18.5)	2013 (19.5)	667 (20.3)	3150 (25.4)	1204 (19.4)	5163 (22.7)
Northwest	4824 (16.7)	301 (10.4)	1984 (19.2)	321 (9.8)	2218 (17.9)	622 (10.0)	4202 (18.5)

Abbreviations: ILI, influenza-like illness; IQR, interquartile range; LAIV, live, attenuated influenza vaccine; TIV, trivalent inactivated influenza vaccine.

a Numbers (percentages) for all data except age, which is reported as median (IQR).

Women, officers, those with a potential alcohol problem, those serving in the Navy or Marine Corps (vs Army or Air Force), those residing in the US Southwest (vs each other region) and in a health care occupation (vs combat or other occupation) were less likely than comparison groups to have received LAIV (p<0.0001). Additionally, compared with 2006/2007, those vaccinated in 2008/2009 were more likely to have received LAIV (p = 0.04). Vaccine type did not significantly differ by age, smoking status, exercise level, marital status, or race/ethnicity.

During the 2 seasons, 3936 ILI events were diagnosed, for an overall crude ILI incidence rate of 136.1 cases per 1000 person-seasons. Weekly ILI case volume demonstrated a pattern similar to that of positive influenza isolates in the general population reported by CDC over the same time period [30], [32]. ILI rates for LAIV and TIV were 138.5 and 127.0 cases per 1000 person-seasons, respectively. The ILI rate by vaccine type did not significantly vary by season.

In the univariable model, type of vaccine was not associated with the development of ILI (HR, 1.02; 95% CI, 0.95–1.11) (Table 3). Factors associated with an increased risk of ILI included recent smoking history, younger age, female sex, other race/ethnicity, and health care occupation. Those serving in the Army or Marine Corps (compared with the Air Force), officers, and combat specialists had a lower risk (Table 3).

**Table 3:** Univariable Hazard Risk Ratios for ILI Among US Service Members, 18–49 Years of Age, During 2006/2007 and 2008/2009 Influenza Seasons.

Characteristic	ILI Events	
	No. (%)	HR (95% CI)
Vaccine type		
TIV	787 (12.7)	1.00 (Ref)
LAIV	3149 (13.9)	1.02 (0.95–1.11)
Recent smoking history		
Yes	1123 (14.1)	1.09 (1.02–1.17)^a^
No	2813 (13.4)	1.00 (Ref)
Potential alcohol problem		
Yes	267 (12.0)	0.90 (0.79–1.02)
No	3669 (13.7)	1.00 (Ref)
Exercise level		
Minimal	439 (14.4)	1.05 (0.94–1.16)
Moderate	2782 (13.7)	1.00 (Ref)
High	133 (10.4)	0.79 (0.66–0.95)
Unknown	582 (13.7)	1.00 (0.91–1.09)
Age, y		0.99 (0.98–0.99)^a^
Sex		
Male	2339 (11.1)	1.00 (Ref)
Female	1597 (20.1)	1.84 (1.72–1.96)^a^
Race/ethnicity		
White	2823 (13.4)	1.00 (Ref)
Black	486 (13.4)	1.00 (0.91–1.10)
Hispanic	290 (14.0)	1.08 (0.95–1.22)
Other/unknown	337 (15.7)	1.17 (1.04–1.32)^a^
Marital status		
Married	2420 (13.6)	1.00 (Ref)
Other	1516 (13.7)	1.02 (0.96–1.09)
Service branch		
Air Force	2363 (16.5)	1.00 (Ref)
Army	1047 (11.1)	0.74 (0.68–0.79)^a^
Marine Corps	197 (6.6)	0.43 (0.37–0.50)^a^
Navy	329 (14.6)	0.90 (0.80–1.02)
Military rank		
Enlisted	3140 (14.3)	1.00 (Ref)
Officer	796 (11.4)	0.77 (0.71–0.84)^a^
Occupation		
Combat	531 (10.2)	0.76 (0.69–0.84)^a^
Health care	783 (18.3)	1.28 (1.18–1.39)^a^
Other	2622 (13.5)	1.00 (Ref)
US geographic area		
Southwest	1203 (13.8)	1.00 (Ref)
Southeast	1192 (13.3)	0.98 (0.90–1.06)
Northeast	832 (13.1)	0.92 (0.84–1.00)
Northwest	709 (14.7)	1.04 (0.95–1.15)
Influenza season		
2006–2007	1860 (14.1)	1.00 (Ref)
2008–2009	2076 (13.2)	1.07 (1.00–1.14)^a^

Abbreviations: CI, confidence interval; HR, hazard ratio; ILI, influenza-like illness; LAIV, live, attenuated influenza vaccine; TIV, trivalent inactivated influenza vaccine.

a *p*<0.05.

In the final multivariate model, adjusted for all significant covariates and using average values for health behaviors in vaccine type interaction terms, the risk of ILI did not significantly differ between LAIV and TIV (HR, 0.98; 95% CI, 0.90–1.06) (Table 4). In the final adjusted model, the relative effectiveness of the 2 vaccine types did not differ by smoking status (*p* = 0.10), alcohol status (*p* = 0.21), or activity level (*p* = 0.11). In addition, ILI risk did not significantly differ by vaccine type among smokers (n = 7955) (HR, 1.09; 95% CI, 0.94–1.27), nonsmokers (n = 20,974) (HR, 0.94; 95% CI, 0.85–1.03), those with a potential alcohol problem (n = 2221) (HR, 1.19; 95% CI, 0.86–1.63), those without an alcohol problem (n = 26,708) (HR, 0.96; 95% CI, 0.89–1.05), those reporting a high exercise level (n = 1283) (HR, 1.12; 95% CI, 0.71–1.76), and those reporting a minimal exercise level (n = 3039) (HR, 1.08; 95% CI, 0.85–1.36) in the model adjusting for sociodemographic factors and the other adverse health behaviors of interest. Similar findings resulted from examining health behaviors by vaccine type for each season separately.

**Table 4:** Risk of ILI by Vaccine Type and Health Behavior During Two Influenza Seasons (2006/2007, 2008/2009).

				Vaccine Type							
				LAIV			TIV				
	Total Study Population			All	ILI Diagnosis		All	ILI Diagnosis			
	N	ILI	%	N	N	%	N	N	%	*p*	HR (95% CI)^a^
Overall	28,929	3936	13.61	22,734	3149	13.85	6195	787	12.70		0.98 (0.90–1.06)
Recent smoking history										0.099	
Nonsmoker	20,974	2813	13.41	16,496	2228	13.51	4478	585	13.06		0.94 (0.85–1.03)
Smoking history	7955	1,123	14.12	6238	921	14.76	1717	202	11.76		1.09 (0.94–1.27)
Possible alcohol problems										0.209	
No drinking problem	26,708	3669	13.74	21,079	2933	13.91	5629	736	13.08		0.96 (0.89–1.05)
Possible drinking problem	2221	267	12.02	1655	216	13.05	566	51	9.01		1.19 (0.86–1.63)
Exercise level										0.114	
Moderate	20,345	2782	13.67	16,032	2209	13.78	4313	573	13.29		0.92 (0.84–1.01)
Minimal	3039	439	14.45	2334	348	14.91	705	91	12.91		1.08 (0.85–1.36)
High	1283	133	10.37	1007	108	10.72	276	25	9.06		1.12 (0.71–1.76)
Unknown	4262	582	13.66	3361	484	14.40	901	98	10.88		1.19 (0.96–1.48)

Abbreviations: CI, confidence interval; HR, hazard ratio; ILI, influenza-like illness; LAIV, live, attenuated influenza vaccine; TIV, trivalent inactivated influenza vaccine.

a Hazard ratio for each health behavior stratum is evaluated at the average level of other behaviors in the study population and adjusted for all other demographic covariates.

## Discussion

This study demonstrated that the relative effectiveness of LAIV versus TIV in preventing ILI among young adults did not vary by health-related behaviors (tobacco, alcohol use, or exercise level) using data from the 2006/2007 and 2008/2009 influenza seasons. These findings are in accordance with current recommendations regarding influenza vaccine from the Advisory Committee on Immunization Practices [44] and indicate that tailoring vaccine type recommendations to these specific health behavior groups is not warranted.

The effectiveness of the influenza vaccine depends on the recipient's immunocompetence, the similarity of vaccine strains to circulating strains in a population (ie, the “match”), the outcome measured, and possibly the mode of vaccine delivery. The role of immunocompetence, since it may be modulated by particular health behaviors, and its interaction with the mode of vaccine delivery were explored by controlling for both the vaccine match (by selecting 2 well-matched seasons) and the outcome measured (ILI). Several health behaviors that may have differential effects on vaccine effectiveness against influenza-related events were examined.

Tobacco and alcohol misuse have been associated with a higher risk for influenza and related complications [11], [12], hence data on the optimal vaccine type in this group are important for patient care and public health strategies. Tobacco use is known to result in respiratory tract (impaired ciliary action) and immune (altered humoral and cell-mediated immunity) dysfunction from exposure to carcinogens, toxins, and lipopolysaccharide [1]. Further, a tobacco dose-related decrease in salivary IgA levels among smokers, a decrease in serum IgG, and alterations in immune cell counts (including lymphocytes, monocytes, B cells, and helper and suppressor T cells) have been described [1]. Hence, impaired mucosal immunity among smokers offers a biologically plausible reason for smokers to respond differently to LAIV (administered to the nasal mucosa) compared with TIV. Prior to this study, a single study had found a significant interaction between cigarette smoking and influenza vaccination by circulating influenza antibodies [45], however, no difference in protection against clinical influenza was found. Overall, these data and the current study findings do not support a differential response by vaccine type among smokers and nonsmokers.

Regarding excessive alcohol use, resultant negative cellular and innate immune system effects, including weakened antiviral defense mechanisms, have been described [2]–[4], [6]–[9]. The antigen-presenting function of innate immune cells (eg, monocytes and macrophages) may be inhibited by alcohol exposure, and both T-cell proliferation and the distribution and functions of memory T cells and natural killer cells altered. Despite these potential immunologic changes, we did not find a differential response by vaccine type among problem drinkers.

Transient immunosuppression has been documented following intense and/or prolonged physical activity, though baseline disease rates have not been shown to differ between athletes and nonathletes [19]–[21], and athletes typically report decreased rates of infection on surveys [19]. Specific immune system effects of exercise such as low salivary IgA associated with intense exercise in elite athletes have been documented and may vary according to an individual's conditioning [20], [21]. However, studies have found that 2–4 weeks after prolonged intensive exercise, individuals mount a normal antibody response to vaccination (tetanus, diphtheria, pneumococcus) [22]–[24]. It seems plausible that altered immunity could also appear with a sedentary lifestyle. Boosting of immunity and reduction in the number of days reporting upper respiratory symptoms among those engaging in exercise compared with sedentary controls have been described [19], [27]. Although, a difference in efficacy conferred by LAIV compared with TIV might be hypothesized among groups with minimal or high exercise levels, we found no differences in ILI by vaccine type.

Overall, no difference in risk of ILI between LAIV and TIV recipients by each of the health behaviors of interest was found, likely reflecting the lack of effect on vaccine responses. It is also possible that the behavior categorization did not adequately separate those more likely to experience immune system effects due to their behavior from those at low risk of such effects; however, the military does contain sufficient numbers of persons with varying health behaviors, and the evaluation of more extreme behaviors may be less generalizable to the overall population.

One methodological limitation of this study is that the outcomes measured were based on ICD-9 codes rather than laboratory-confirmed influenza diagnosis; however, the codes used have been previously shown to correlate with viral culture or polymerase chain reaction-confirmed influenza events [38], [39], [46]. Also, illnesses that did not result in a health care visit within the military system could not be captured. Health behavior data were self-reported on a questionnaire and may not reflect the actual behaviors at the time of vaccine receipt. Since this study examined the potential associations between exposures and the effectiveness of the different vaccine types, by design it was only able to evaluate the apparent relative effectiveness of each vaccine. Lastly, this study included only a subset of adults serving in the US military; hence, data may not be representative of the entire military population. Further, although service members reflect a fairly broad spectrum of socioeconomic status and geographic distribution, additional research may be warranted to examine differential vaccine type effects on influenza outcomes among civilian groups with other health behavior profiles.

To the authors' knowledge, this is the first study to examine the effects of particular health behaviors on the relationship between vaccine type and influenza outcome. Strengths of this study include its robust size with nearly 30,000 vaccination events evaluated. In addition, the study utilized self-reported health behavior data proximate to vaccine receipt in a large population with reliable health care encounter capture. Further, military vaccine records used were comprehensive and highly reliable. Finally, the ILI rate (13.6%, n>3,000) allowed for robust measurement of the effects of health behavior factors.

In summary, LAIV and TIV have similar effectiveness in preventing ILI events among healthy 18- to 49-year-olds who are smokers, have potential alcohol problems, or endorse minimal or high exercise levels. These data suggest that clinicians and public health practitioners need not tailor vaccine regimens according to the particular health behavior groups studied here, and instead should focus vaccination efforts on simply ensuring widespread vaccination.
